# Metabolomic Responses of Maize Shoots and Roots Elicited by Combinatorial Seed Treatments With Microbial and Non-microbial Biostimulants

**DOI:** 10.3389/fmicb.2020.00664

**Published:** 2020-05-06

**Authors:** Youssef Rouphael, Luigi Lucini, Begoña Miras-Moreno, Giuseppe Colla, Paolo Bonini, Mariateresa Cardarelli

**Affiliations:** ^1^Department of Agricultural Sciences, University of Naples Federico II, Portici, Italy; ^2^Department for Sustainable Food Process, Research Centre for Nutrigenomics and Proteomics, Università Cattolica del Sacro Cuore, Piacenza, Italy; ^3^Council for Agricultural Research and Economics-Research Centre for Genomics and Bioinformatics, Fiorenzuola d’Arda, Italy; ^4^Department of Agriculture and Forest Sciences, University of Tuscia, Viterbo, Italy; ^5^NGA Laboratory, Tarragona, Spain; ^6^Consiglio per la Ricerca in Agricoltura el’ Analisi dell’Economia Agraria, Centro di Ricerca Orticoltura e Florovivaismo, Pontecagnano Faiano, Italy

**Keywords:** protein hydrolyzate, mycorrhiza, phytohormones, plant metabolomics, *Trichoderma*, *Zea mays* L.

## Abstract

Microbial and non-microbial plant biostimulants have been successfully used to improve agriculture productivity in a more sustainable manner. Since the mode of action of biostimulants is still largely unknown, the present work aimed at elucidating the morpho-physiological and metabolomic changes occurring in maize (*Zea mays* L.) leaves and roots following seed treatment with (i) a consortium of two beneficial fungi [arbuscular mycorrhizal fungi (AMF) and *Trichoderma koningii* TK7] and rhizobacteria, (ii) a protein hydrolyzate-based biostimulant (PH) alone, or (iii) in combination with a consortium of *T. koningii* TK7 and rhizobacteria. The application of PH alone or in combination with *Trichoderma* elicited significant increases (+16.6%) in the shoot biomass compared to untreated maize plants, whereas inoculation with AMF + *Trichoderma* elicited significant increases in root dry biomass (+48.0%) compared to untreated plants. Distinctive metabolomic signatures were achieved from the different treatments, hence suggesting that different molecular processes were involved in the plants response to the biostimulants. The metabolic reprogramming triggered by the treatments including the protein hydrolyzate was hierarchically more pronounced than the application of microorganisms alone. Most of the differential metabolites could be ascribed to the secondary metabolism, with phenylpropanoids and terpenes being the most represented compounds. The application of PH triggered an accumulation of secondary metabolites, whereas the opposite trend of accumulation was seen in the case of microorganisms alone. The increase in biomass could be related to two processes, namely the modulation of the multilayer phytohormone interaction network and a possible increase in nitrogen use efficiency via the GS-GOGAT system.

## Introduction

Nowadays, agriculture is facing new and concurrent challenges such as boosting crop productivity and coping with food insecurity. Considering that the global population will reach 10 billion by 2055 the environmental impact of agriculture has to be minimized and natural resources (i.e., soil quality and water) have to be preserved for future generations, both sustainability and low production costs are required to remain competitive in a globalized economy (FAO, 2009). Maize (*Zea mays* L.) has gained economic significance at a global level, contributing about 12.4% of the world’s food demand (38% of cereals) and ranking first in production volume worldwide (1,135 million tons) (FAO, 2017). Enhancing resource use efficiency (e.g., water and nutrients) through novel, sustainable and eco-friendly strategies, is an urgent need to secure yield stability and food security while preserving soil quality and providing new business opportunities for farmers (Searchinger et al., 2018).

An eco-friendly, sustainable and innovative method that is able to tackle the upcoming challenges is the incorporation of biostimulant technology in the cropping system, especially biostimulants of plant origin (Chiaiese et al., 2018; Rouphael et al., 2018c). In a recent EU Regulation, “plant biostimulant” has been defined as an EU fertilizing product which is applied to crop plants or rhizospheres with the aim of modulating plant physiological functions and of improving crop productivity, efficiency of nutrient use, quality of crop products and abiotic stresses tolerance (EU, 2019). Plant biostimulants by this definition include several substances with bioactive properties: humic and fulvic acids, protein hydrolyzates, seaweed extracts, plant extracts and silicon, as well as plant growth promoting microorganisms: arbuscular mycorrhizal fungi (AMF), *Trichoderma*, and plant growth promoting rhizobacteria (PGPR) (Calvo et al., 2014; Battacharyya et al., 2015; Canellas et al., 2015; Colla and Rouphael, 2015; Colla et al., 2015a, 2017b; du Jardin, 2015; Rouphael and Colla, 2018, 2020).

Arbuscular mycorrhizal fungi and *Trichoderma* represent two major classes of beneficial microbes (López-Bucio et al., 2015; Rouphael et al., 2015). The biostimulant action of these endophytic fungi under both favorable and stressful soil or environmental conditions has been associated with several putative mechanisms including: (i) the production of key enzymes such as phosphatases and/or release of small peptides, volatiles, and active metabolites that have hormone-like activity, (ii) enhancing photosynthetic efficiency and water relations, (iii) the promotion of nitrate, phosphate and ammonia transporters, (iv) the accumulation of osmoprotectants and antioxidants, and (v) the modulation of plant root architecture through the increase of root length, density and branching, resulting in enhanced nutrient uptake (P, Fe, Mn, and Zn) (Contreras-Cornejo et al., 2009; Woo et al., 2014; López-Bucio et al., 2015; Rouphael et al., 2015; Fiorentino et al., 2018; Lucini et al., 2019; Saia et al., 2019, 2020).

Another prominent category of non-microbial plant biostimulants that has demonstrated beneficial effects on shoot and root stimulation, similar to those exerted by microbial-based biostimulants, is represented by protein hydrolyzates. In particular they contain a mixture of free amino acids and peptides obtained via the chemical or enzymatic partial hydrolysis of protein sources from either animal or vegetal origin (Calvo et al., 2014; Haplern et al., 2015). Direct and indirect modes of action underlying the biostimulant activity of plant- or animal-based protein hydrolyzates include: (i) the stimulation of C and N metabolism by triggering key enzymes, (ii) the induction of hormone-like activities, in particular those of auxins and gibberellins, (iii) the stimulation of secondary metabolism by increasing antioxidant capacity, (iv) the modulation of root growth which can consequently result in a ‘*nutrient acquisition response*’ improving resource use efficiency (Schiavon et al., 2008; Ertani et al., 2009, 2013, 2017; Colla et al., 2015a,b, 2017a,b; Rouphael et al., 2017, 2018b; Sestili et al., 2018).

For field crop species such as maize, biostimulants (and in particular the microbial ones) are applied as a seed treatment or directly onto plant and soil. However, seed treatment has been proven to be an economical and efficient tool to introduce non-microbial and microbial biostimulants in the soil rhizosphere, compared to foliar spray or substrate/soil drench where a high quantity of the product is required (Tavares et al., 2013; Colla et al., 2015b).

Despite the significant advancements made in the last decade in terms of studying the effects of plant biostimulants on a broad spectrum of field and horticultural crops, there are two main bottlenecks that hamper scientists, private industries and farmers from extensively implementing plant biostimulants into agronomic practices. Firstly, an increase in knowledge about the molecular and physiological mechanisms underlying biostimulant action could substantially support and facilitate the diffusion of these products in the agricultural sector. Second, an increased awareness and knowledge about the combined application of microbial and/or non-microbial plant biostimulants may represent a valuable solution to render agriculture more resilient and sustainable.

From this perspective, the omics sciences, and in particular untargeted metabolomics, are considered to be a powerful tool to reveal the effects of biotic and abiotic factors on plant physiology (Tenenboim and Brotman, 2016). Metabolomics can help to shed light onto the biochemical processes involved in plant response to biostimulants (Bernardo et al., 2019; Wu et al., 2019). Therefore, the current research aims to overcome the above-reported bottlenecks by investigating the changes in shoot and root biomass and partitioning together with the metabolic reprogramming elicited on maize by either endophytic fungi (*Trichoderma koningii* TK7 and mycorrhiza: *Rhizoglomus irregulare* BEG72 and *Funneliformis mosseae* BEG 234), a legume-derived protein hydrolyzate, alone or as a combination of the both legume-derived protein hydrolyzate with *T. koningii* TK7. Finally, the insights from this work can improve the poor knowledge of the mode(s) of action and can address the development and optimization of plant biostimulants.

## Materials and Methods

### Tested Crop, Greenhouse Growth Conditions, and Experimental Setup

The trial was conducted in the 2016 growing season in a polyethylene greenhouse located at the Experimental Farm ‘Nello Lupori’ of Tuscia University, central Italy (latitude 42°25′ N, longitude 12°08′ E, altitude 310 m). Inside the polyethylene greenhouse, ventilation was provided automatically when the air temperature exceeded 26°C, and light was provided only by natural solar radiation. During the experiment, daily mean values of solar radiation at crop level ranged from 16.1 to 22.3 MJ m^–2^. The mean air temperature and relative humidity inside the greenhouse were 20°C and 60%, respectively. The trial was performed on maize (*Zea mays* L.) cultivar ‘PR36B08’ (Pioner, Gadesco-Pieve Delmona, Italy) belonging to FAO class 300.

Seeds of maize were surface sterilized with a solution containing 80% of ethanol. After sterilization (10 min), the seeds were washed two times with sterile distilled water. On May 4, maize seeds were sown in plastic pots (diameter of 18 cm) filled with 4.0 L of fluvial sand at a rate of five seeds per pot. Fluvial sand was previously washed with distilled water using 5 L of water for each liter of sand, and then sterilized in an autoclave twice to kill spores of bacteria and fungi. The experiment included the following four treatments:

(1) seed treatment with 3 g of a consortium of arbuscular mycorrhizal fungi (AMF), *T. koningii* TK7 and rhizosphere bacteria (‘Covenant,’ produced by Atens, Agrotecnologías Naturales, S. L., Tarragona, Spain, containing 230 spores g^–1^ of *Rhizoglomus irregulare* BEG72, 230 spores g^–1^ of *Funneliformis mosseae* BEG 234, 3 × 10^8^ Colony-forming unit [CFU] g^–1^ of *T. koningii* TK7, 4 × 10^7^ CFU g^–1^ of rhizosphere bacteria such as *Bacillus megaterium* MHBM77) per 1 kg of seed;
(2) seed treatment with a solution containing 0.3 g of a consortium of *T. koningii* TK7 and rhizosphere bacteria such as *Bacillus megaterium* MHBM77 (‘Covenant Trichoderma,’ produced by Atens, Agrotecnologías Naturales, S. L., Tarragona, Spain, containing 2 × 10^9^ CFU g^–1^ of *T. koningii* TK7, 1 × 10^7^ CFU g^–1^ of rhizosphere bacteria such as *Bacillus megaterium* MHBM77) plus 0.45 ml of a protein hydrolyzate-based biostimulant (PH) (‘Coveron Stim’ produced by Italpollina s.p.a, Rivoli Veronese, Italy, containing 70 g kg^–1^ of organic N) per 1 kg of seed;
(3) seed treatment with 0.45 ml of a protein hydrolyzate-based biostimulant (‘Coveron Stim’) per 1 kg of seed;
(4) untreated control.

The protein hydrolyzate included in the ‘Covenant’ and ‘Coveron Stim’ products was a legume-derived protein hydrolyzate obtained through enzymatic hydrolysis of proteins derived from legume seeds. It contains 50 g kg^–1^ of N as free amino acids, and soluble peptides. The aminogram of the product in g kg^–1^ was: Ala (12), Arg (18), Asp (34), Cys (3), Glu (54), Gly (12), His (8), Ile (13), Leu (22), Lys (18), Met (4), Phe (15), Pro (15), Thr (11), Trp (3), Tyr (11), Val (14). All three products contain a green natural colorant for verifying the uniformity of product distribution on seed surface.

Seed treatments were performed with a seed-treatment machine able to automatically spray the seed surface with a water suspension/solution containing the products at a rate of 10 ml per kg of seed. Treatments were arranged in a randomized block design with three replicates. Each experimental unit consisted of six pots. Besides the required pots (18 pots per treatment), 10 extra pots per treatment were also prepared. One week after the initial emergence of seedlings, pots having less than four plants were discarded.

Plants were fertirrigated starting 1 week after sowing. The basic nutrient solution used was a modified Hoagland and Arnon formulation having the following macro and micro mineral composition: 7.0 mM N–${\text{NO}}_{3}^{-}$, 1.5 mM S, 0.2 mM P, 2.7 mM K, 5.5 mM Ca, 1.5 mM Mg, 20.0 μM Fe, 9.0 μM Mn, 0.3 μM Cu, 1.6 μM Zn, 20.0 μM B, and 0.3 μM Mo. The nutrient solution was prepared using de-mineralized water. The electrical conductivity and pH of the nutrient solution were 1.8 dS m^–1^ and 6.0, respectively.

### Biomass Determination, Partitioning, and SPAD Index Measurement

At 16 days after sowing (May 19), all maize plants per experimental plot (i.e., the replicates) were harvested and the shoots (sum of leaves and stems) were separated from the roots. Shoots and roots were dried in a forced-air oven at 70°C for 72 h until constant weight, then the shoots and roots dry matter content were recorded. The root-to-shoot ratio was also calculated.

Plant Analysis Development (SPAD) index (i.e., a non-destructive measurement of chlorophyll content) was measured on undamaged maize leaves by means of a portable SPAD-502 chlorophyll meter (Konica-Minolta, Tokyo, Japan). Ten measurements were conducted on randomly picked maize leaves per experimental plot, then averaged to a single SPAD value for each replicate as described by Kumar et al. (2015).

### Sampling and Untargeted Metabolomic Analysis

The first expanded maize leaf and terminal roots samples were collected from two plants per experimental plot (i.e., replicate) from each of the four tested treatments. Samples of maize leaf and roots (six samples of leaves and sx samples of roots per each treatment) were ground with liquid nitrogen using pestle and mortar, and then extracted as previously reported (Rouphael et al., 2018b). Briefly, an aliquot (1.0 g) was extracted in 10 mL of 0.1% HCOOH in 80% aqueous methanol using an Ultra-Turrax (Ika T-25, Staufen, Germany). The extracts were centrifuged (12000 × *g*) and the untargeted metabolomic screening was carried out using an UHPLC liquid chromatography system and a quadrupole-time-of-flight mass spectrometer equipped with an electrospray ionization source (UHPLC/Q-TOF), according to a previously reported set up (Pretali et al., 2016). In more detail, a 1290 LC system was coupled to a G6550 quadrupole-time-of-flight mass spectrometer (Agilent Technologies Santa Clara, CA, United States). Chromatographic separation was achieved in reverse phase mode, using a C18 column (100 mm × 2.1 mm, 1.8 μm) and a binary gradient consisting of water and methanol (from 5 to 90% organic in 34 min) with a flow rate of 200 μL min^–1^. The mass spectrometer operated in the positive polarity and in SCAN mode (range of 100–1200 m/z in extended dynamic range settings).

Compound annotation was achieved by combining both the monoisotopic accurate mass and isotopic pattern (i.e., isotope spacing and ratio), adopting a mass accuracy tolerance of <5 ppm and using the software Profinder B.07 (Agilent technologies) and a database exported from PlantCyc 9.6 (Plant Metabolic Network^1^). The annotation strategy corresponded to Level 2 (putatively annotated compounds) of COSMOS Metabolomics Standards Initiative^2^. A filter-by-frequency algorithm was then applied, retaining only those compounds present in 75% of replicates within at least one treatment.

### Statistics and Chemometrics

The statistical analysis was carried out using IBM SPSS Statistics 20 (Chicago, IL, United States). The plant biomass and partitioning, as well as SPAD index, were subjected to one-way analysis of variance (ANOVA). Mean values were separated according to a Duncan test with *P* = 0.05.

Interpretation of the metabolomic analysis was initially carried out using Mass Profiler Professional 12.6 (Agilent Technologies) for log2 transformation of compound abundance, normalization at the 75th percentile, and baselining against the median of each compound in the dataset. Thereafter, hierarchical cluster analysis (Euclidean distance, Ward’s linkage) was performed to investigate in an unsupervised manner the relatedness across treatments. The dataset was then imported into SIMCA 13 software (Umetrics, Malmö Municipality, Sweden) and elaborated through supervised orthogonal projection to latent structures discriminant analysis (OPLS-DA). The OPLS-DA model was cross validated (CV-ANOVA), inspected for outliers (Hotelling’s T2), and thereafter model parameters (degree of correlation and prediction ability, R2Y and Q2Y respectively) were recorded. Overfitting was excluded through a permutation test (*n* = 100) and discriminant compounds selected by variables importance in projection (VIP) in the OPLS predictive model. The VIP score was calculated as a weighted sum of the squared correlations between the OPLS-DA components and the original variables. Compounds possessing a score > 1.32 were selected as discriminants. To gain a more in-depth knowledge about the individual effect of each biostimulant on the plant physiology Volcano analysis was also performed (*P* < 0.01, Bonferroni multiple testing correction; fold-change FC ≥ 2) and the differential compounds were exported to the PlantCyc pathway Tools software (Karp et al., 2010) to highlight the metabolic pathways and processes involved in plant response to treatments.

## Results

### Biostimulant Action of Microbial and Non-microbial Biostimulants on Growth Responses and SPAD Index

The results concerning plant biomass and partitioning as well as SPAD index in relation to microbial (AMF + *Trichoderma*) and non-microbial [protein hydrolyzate (PH) biostimulants alone or in combination with *Trichoderma* are presented in Table 1. Concerning the effects of biostimulant application on growth responses, our findings showed that the application of PH alone or in combination with *Trichoderma* elicited significant increases (+16.6%) in the shoots compared to untreated maize plants, whereas inoculation with AMF + *Trichoderma*, exhibited intermediate values. Regarding the root dry biomass, the inoculation with AMF + *Trichoderma* elicited significant increases (+48.0%) compared to untreated plants, whereas both PH and PH + *Trichoderma* combinations exhibited intermediate values (average +25% compared to the control treatment) (Table 1). The co-inoculation of maize plants with AMF and *T. koningii* TK7 enhanced the root-to-shoot ratio and SPAD index compared to the other three treatments (i.e., untreated or treated with PH and PH + *Trichoderma*; Table 1). Finally, the highest values of shoot and root dry matter content were recorded in PH and in both PH or PH + *Trichoderma* treatments, respectively (Table 1).

**TABLE 1:** Effect of seed treatments with a consortium of arbuscular mycorrhizal fungi (AMF), *Trichoderma koningii* TK7, and rhizosphere bacteria or with a consortium of *Trichoderma koningii* TK7 and rhizosphere bacteria (Tricho) plus a protein hydrolyzate-based biostimulant (PH), or with a protein hydrolyzate-based biostimulant (PH), on dry weight and dry matter of shoots and roots, root-to-shoot ratio and SPAD index in maize plants.

Treatment	Shoot		Roots		Root to shoot	SPAD index
	Dry weight (g plant^–1^)	Dry matter (%)	Dry weight (g plant^–1^)	Dry matter (%)		
Control	131.5b	10.9ab	29.8c	7.7b	0.21b	27.7b
AMF + Tricho	141.0ab	9.7b	44.1a	8.4ab	0.33a	30.8a
PH	155.0a	11.7a	36.6b	8.9a	0.24b	28.0b
PH + Tricho	151.8a	10.9ab	37.9b	8.8a	0.25b	28.6ab
Significance	*	*	***	*	**	*

*, **, ***, significant at P ≤ 0.05, 0.01, and 0.001, respectively. Different letters within each column indicate significant differences according to Duncan’s multiple-range test (P = 0.05).

### Implications of Microbial and Non-microbial Seed Tanning on the Metabolomic Profiling of Maize Leaves and Roots

In order to understand the effect of the different microbial and non-microbial biostimulants on maize at a molecular level, an untargeted metabolomic approach based on UHPLC-QTOF mass spectrometry was carried out. Overall, the metabolomic analysis lead us to putatively annotate more than 3,600 compounds in leaves and/or roots (Supplementary Table 1) that allowed us to discriminate the four treatments based on their metabolomic signatures.

**FIGURE 1:**
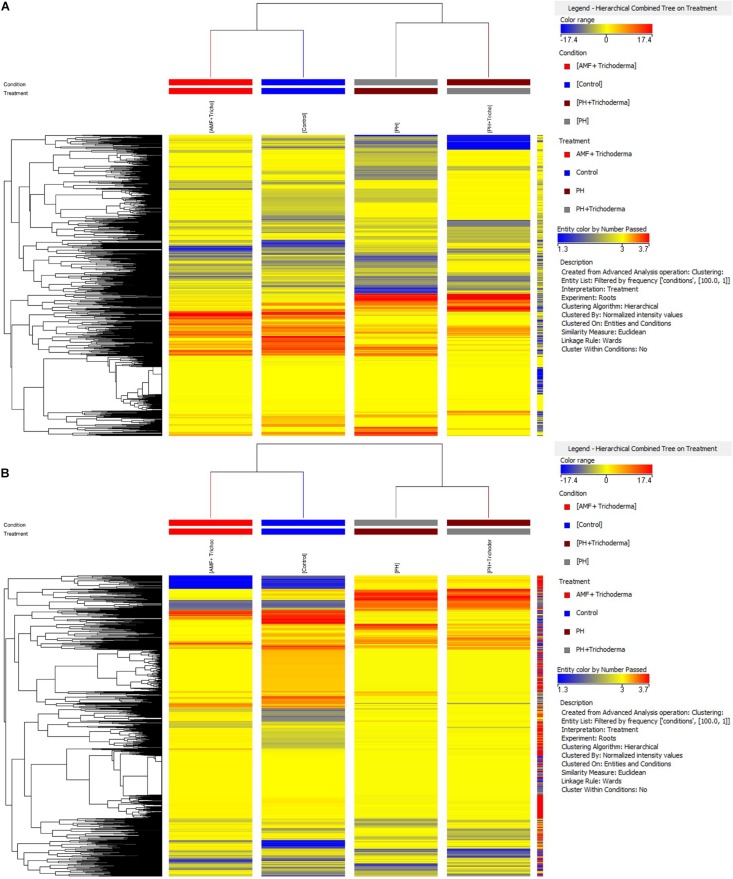
Unsupervised hierarchical cluster analysis carried out from UHPLC-ESI/QTOF-MS metabolomic analysis of leaves **(A)** and roots **(B)** of maize plants treated with a consortium of arbuscular mycorrhizal fungi (AMF), *Trichoderma koningii* TK7, and rhizosphere bacteria or with a consortium of *Trichoderma koningii* TK7 and rhizosphere bacteria (Tricho) plus a protein hydrolyzate-based biostimulant (PH), or with a protein hydrolyzate-based biostimulant (PH). The fold-change based heat map was used to build hierarchical clusters (linkage rule: Ward; distance: Euclidean).

In particular, an unsupervised hierarchical cluster analysis was first performed to identify similarities/dissimilarities among the treatments based on their metabolic profiles. The fold-change based heat map grouped the treatments in two main clusters (Figure 1). The combined treatment with microorganisms showed a less pronounced effect on both roots and leaves, as compared to the PH application, and considering that the AMF + *Trichoderma* treatment clustered together with the untreated control. However, a second well-separated cluster including the PH treatment and the combined PH + *Trichoderma* treatment was observed (Figure 1).

**FIGURE 2:**
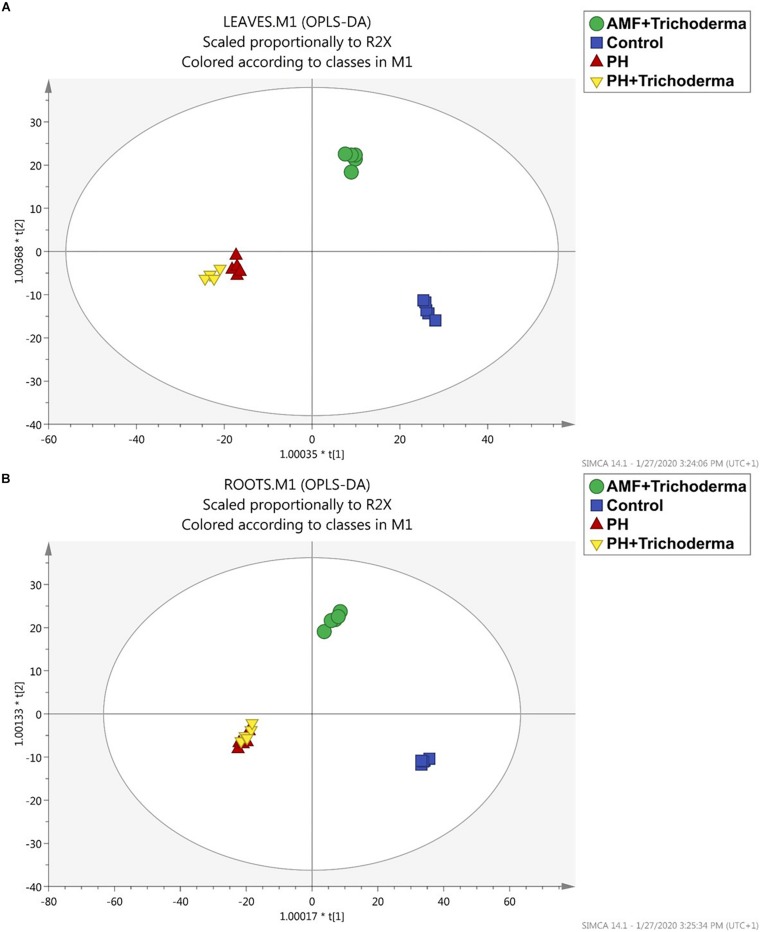
Orthogonal projection to latent structures discriminant analysis (OPLS-DA) supervised modeling of leaves **(A)** and roots **(B)** of maize plants treated with a consortium of arbuscular mycorrhizal fungi (AMF), *Trichoderma koningii* TK7, and rhizosphere bacteria or with a consortium of *Trichoderma koningii* TK7 and rhizosphere bacteria (Tricho) plus a protein hydrolyzate-based biostimulant (PH), or with a protein hydrolyzate-based biostimulant (PH). The metabolomic dataset produced through UHPLC-ESI/QTOF-MS was Pareto scaled and then used for the multivariate OPLS-DA modeling.

After the unsupervised analysis, a supervised multivariate analysis of the metabolomics-based data was produced to better highlight the differences between treatments and identify discriminant compounds. Indeed, OPLS discriminant analysis was more effective in separating all the biostimulant-treated maize plants from the untreated control (Figure 2). The OPLS model indicators emphasized the predictivity of the model with R2Y (the goodness-of-fit) = 0.98 and 0.99 and Q2Y (goodness-of-prediction) = 0.52 and 0.64 for leaves and roots, respectively. The first latent vector could discriminate between the PH-containing treatment and the other groups, while the second vector accounted for the differences between the combined treatment with microbial biostimulants (AMF + *Trichoderma*) and the control treatment. Although the application of microorganisms induced a distinctive metabolic reprogramming, the PH-containing treatment showed the highest effect since the replicates were found to be completely separated from the other treatments in both leaves and roots. However, no separation was achieved between the PH treatment alone and the combined PH + *Trichoderma* treatment, thus strengthening the hypothesis of a hierarchically more pronounced effect of the PH-based products.

Overall, 216 compounds were recorded as discriminant in leaves by VIP analysis (Supplementary Table 2). Isoprenoids and phenylpropanoids were the most represented class of metabolites. Among isoprenoids, our results highlighted diterpenoids related to hormones biosynthetic such as kauralexin andent-kaurenal, as well as gibberellins, sesquiterpenes, and carotenoids. However, 133 compounds were pointed out as markers by VIP analysis in roots (Supplementary Table 3). Among these compounds, we underlined the presence of hormones including gibberellins, brassinosteroids and abscisic acid, together with jasmonate-related compounds.

**FIGURE 3:**
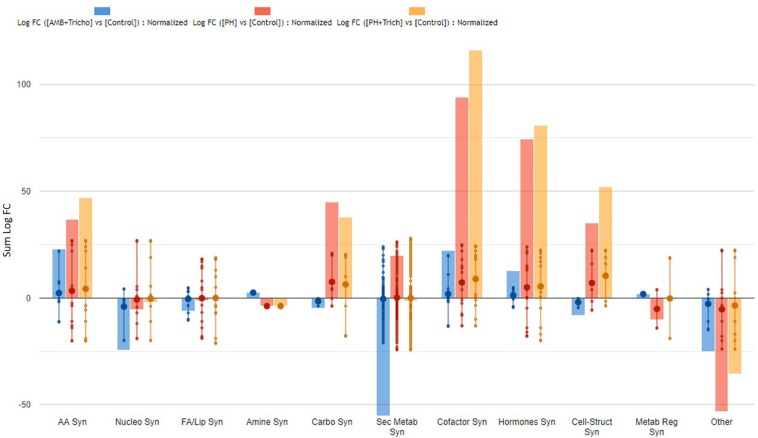
Biosynthesis processes which take place in leaves of maize plants treated with a consortium of arbuscular mycorrhizal fungi (AMF), *Trichoderma koningii* TK7, and rhizosphere bacteria or with a consortium of *Trichoderma koningii* TK7 and rhizosphere bacteria (Tricho) plus a protein hydrolyzate-based biostimulant (PH), or with a protein hydrolyzate-based biostimulant (PH). The metabolomic dataset produced through UHPLC-ESI/QTOF-MS was subjected to a Volcano Plot analysis (*P* < 0.01, fold-change > 1.2) and differential metabolites were loaded into PlantCyc Pathway Tool (https://www.plantcyc.org/). The x-axis represents each set of subcategories while the y-axis corresponds to the cumulative fold-change.

**FIGURE 4:**
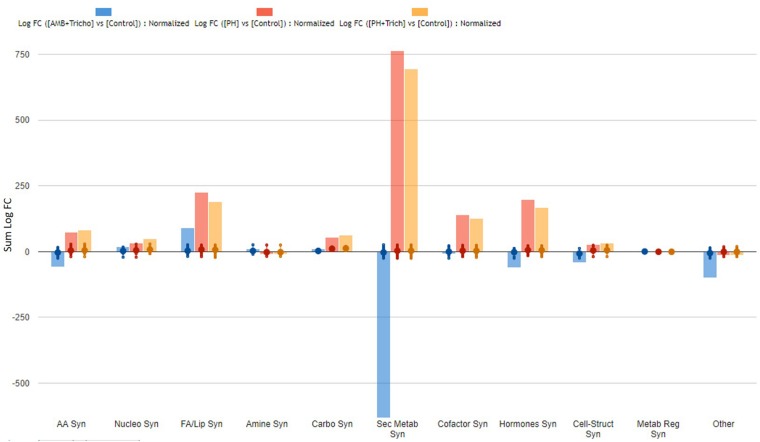
Biosynthesis processes which take place in roots of maize plants treated with a consortium of arbuscular mycorrhizal fungi (AMF), *Trichoderma koningii* TK7, and rhizosphere bacteria or with a consortium of *Trichoderma koningii* TK7 and rhizosphere bacteria (Tricho) plus a protein hydrolyzate-based biostimulant (PH), or with a protein hydrolyzate-based biostimulant (PH). The metabolomic dataset produced through UHPLC-ESI/QTOF-MS was subjected to a Volcano Plot analysis (*P* < 0.01, fold-change > 1.2) and differential metabolites were loaded into PlantCyc Pathway Tool (https://www.plantcyc.org/). The x-axis represents each set of subcategories while the y-axis corresponds to the cumulative fold-change.

The complete list of significant metabolites derived from Volcano analysis was consistent with the outcome of VIP analysis and is provided as Supplementary material (Supplementary Tables 4, 5). Interestingly, secondary metabolism was affected by all treatments in both leaves (Figure 3) and roots (Figure 4). Irrespective from the plant organ considered, the treatment with microorganisms in combination (AMF + *Trichoderma*) showed an effect on secondary metabolism with a general down-regulation, while PH alone or in combination with *Trichoderma* showed an up-regulation of secondary metabolism in leaves (Figure 3). It can be seen that secondary metabolism related compounds were the most involved, since over 150 compounds in leaves were affected by the treatments (Table 2). This PH-mediated response was characterized by the marked increase of phenylpropanoids (up-accumulated following PH application), at the expense of other phytoalexins, in contrast to the microorganism-treated plants. In fact L-phenylalanine, the upstream key compound in the phenylpropanoid biosynthetic pathway, as well as other amino acids and intermediate compounds, were up-regulated in leaves in the presence of PH. The omics viewer built-into PlantCyc allowed the visualization of some other groups of small molecules altered by the treatments including cofactors and prosthetic groups (tetrapyrroles and porphyrins), electron carriers (quinols and quinones), and vitamins. Similar metabolic trends could be observed in both the PH and PH + *Trichoderma* treatments. Although apparently displaying a similar response, the AMF + *Trichoderma* treatment showed a general down-accumulation of secondary metabolism, with several compounds (not affected by PH) being strongly decreased. In addition, the profile of phytohormones was also altered by the treatments. Gibberellin-related compounds were more abundant in the presence of PH alone or in combination with *Trichoderma*, whereas an opposite trend could be observed for abscisic acid. Although less evident, an accumulation of both brassinosteroids and cytokinins could be observed following PH application.

**TABLE 2:** Summarized biosynthesis processes highlighted in leaves of maize plants treated with a consortium of arbuscular mycorrhizal fungi (AMF), *Trichoderma koningii* TK7, and rhizosphere bacteria or with a consortium of *Trichoderma koningii* TK7 and rhizosphere bacteria (Tricho) plus a protein hydrolyzate-based biostimulant (PH), or with a protein hydrolyzate-based biostimulant (PH).

	AMF + *Trichoderma*			PH			PH + *Trichoderma*		
	Number of compounds	Average FC	Sum FC	Number of compounds	Average FC	Sum FC	Number of compounds	Average FC	Sum FC
Amino acid	10	2.3	23.0	11	3.3	36.8	11	4.3	47.0
Nucleosides and Nucleotides	6	–4.1	–24.4	8	–0.7	–5.6	8	–0.3	–2.2
Fatty acid and Lipid	14	–0.4	–6.1	15	–0.1	–1.1	15	0.0	–0.1
Amines and Polyamines	1	2.5	2.5	1	–3.8	–3.8	1	–3.8	–3.8
Carbohydrates 3	−1.6	–4.7	6	7.5	45.1	6	6.3	37.9	
Secondary Metabolites	144	–0.4	–55.5	167	0.3	44.3	167	0.1	23.1
Cofactors, Prosthetic Groups, Electron Carriers	12	1.9	22.4	13	7.2	94.2	13	8.9	116.2
Hormones	10	1.3	12.7	15	5.0	74.4	15	5.4	81.0
Cell structures	4	–2.0	–8.1	5	4.0	35.2	5	10.4	52.0
Metabolic Regulators	1	1.8	1.8	2	–5.1	–10.3	2	–0.2	–0.5
Other biosynthesis	9	–2.8	–25.0	0	–5.3	–53.3	10	–3.5	–35.7

*The metabolomic dataset produced through UHPLC-ESI/QTOF-MS was subjected to a Volcano Plot analysis (P < 0.01, fold-change > 1.2) and differential metabolites were loaded into PlantCyc Pathway Tool (https://www.plantcyc.org/). The average and summed Log fold-changes (Log FC) values, together with the number of compounds involved, is provided for each biosynthetic pathway and for each treatment.*

On the other hand, the treatments seemed to have a more pronounced effect on roots, since more than 500 compounds were found as discriminants by the Volcano analysis (as compared to about 330 compounds significantly affected in leaves) (Supplementary Tables 4, 5). The effect of PH and PH + *Trichoderma* on the root metabolome was very similar (Figure 4) even though plants treated with AMF + *Trichoderma* showed a distinct signature. Consistently with leaves, the biosynthesis plot of PlantCyc showed that secondary metabolism was the most affected by the treatments. Indeed, over 200 metabolites involved in secondary pathways were identified overall (Table 3). The AMF + *Trichoderma* combined treatment caused a general decrease in secondary metabolites while the roots in the presence of PH, alone or in combination, showed an increase in this class of metabolites. Phenylpropanoids, N-containing compounds and terpenoids were the most down-regulated groups in the AMF + *Trichoderma* treatment. In contrast, these compounds together with other phytoalexins and polyketides, were the most up-regulated secondary compounds in the presence of PHs. As noted for leaves, the quinol and quinones pathway was significantly up-regulated in the presence of PH. The metabolic reprogramming induced by the treatments on phytohormones was also more pronounced in roots than leaves. A generalized increase of auxins and a decrease in jasmonates could be observed in all treatments, whereas the opposite trends could be observed when microorganisms were applied alone, compared to the treatments including PH. Gibberellin- and cytokinin-related compounds were more abundant following PH application and less abundant in presence of the microorganisms (AMF + *Trichoderma*), compared to the control. The same trend could be observed for brassinosteroids.

**TABLE 3:** Summarized biosynthesis processes highlighted in roots of maize plants treated with a consortium of arbuscular mycorrhizal fungi (AMF), *Trichoderma koningii* TK7, and rhizosphere bacteria or with a consortium of *Trichoderma koningii* TK7 and rhizosphere bacteria (Tricho) plus a protein hydrolyzate-based biostimulant (PH), or with a protein hydrolyzate-based biostimulant (PH).

	AMF + *Trichoderma*			PH			PH + *Trichoderma*		
	Number of compounds	Average FC	Sum FC	Number of compounds	Average FC	Sum FC	Number of compounds	Average FC	Sum FC
Amino acid	18	–3.2	–58.5	19	4.0	75.3	19	4.4	82.7
Nucleosides and Nucleotides	7	2.5	17.8	7	4.5	31.6	7	7.2	50.6
Fatty acid and Lipid	29	3.1	90.0	29	7.8	225.6	29	6.5	189.5
Amines and Polyamines	4	2.7	10.8	4	–2.2	–8.7	4	–2.2	–8.7
Carbohydrates	4	2.6	10.4	5	10.8	54.0	5	12.4	61.9
Secondary Metabolites	224	–2.8	–621.6	242	3.3	809.2	244	3.0	741.1
Cofactors, Prosthetic Groups, Electron Carriers	43	–0.2	–7.4	44	3.2	141.1	44	2.9	126.5
Hormones	31	–2.0	–62.4	35	5.7	197.9	35	4.8	166.7
Cell structures	6	–7.2	–43.1	6	4.3	26.1	6	5.2	31.3
Metabolic Regulators	3	0.5	1.5	3	–0.7	–2.1	3	–0.7	–2.1
Other biosynthesis	19	–5.3	–100.7	20	–0.7	–13.1	20	–0.7	–14.9

*The metabolomic dataset produced through UHPLC-ESI/QTOF-MS was subjected to a Volcano Plot analysis (P < 0.01, fold-change > 1.2) and differential metabolites were loaded into PlantCyc Pathway Tool (https://www.plantcyc.org/). The average and summed Log fold-changes (Log FC) values, together with the number of compounds involved, is provided for each biosynthetic pathway and for each treatment.*

Finally, fatty acids, amino acids and their intermediate compounds showed an up-accumulation in the presence of PH or PH + *Trichoderma*. Interestingly, glutamate, glutamine and asparagine were accumulated in roots, whereas only asparagine accumulated in leaves following PH application. This may indicate that glutamine synthetase – glutamine oxoglutarate aminotransferase (GS-GOGAT) was involved in the maize response to the treatments including PH, thus suggesting the involvement of nitrogen assimilation and the subsequent export to shoots.

## Discussion

Biostimulants are EU fertilizing products having a beneficial effect on plants in low quantities because they are able to improve one or more of the following characteristics: (i) tolerance to abiotic stress, (ii) nutrient use efficiency, and (iii) quality traits (EU, 2019). These products include both substances and beneficial microorganisms (du Jardin, 2015). Since the mode of action of biostimulants is still largely unknown, the effect of microorganisms (in particular AMF and *T. koningii* TK7) in combination with PHs was investigated in order to shed light onto the molecular and biochemical processes following their application to plants. Diverse responses were found in plants treated with microorganisms alone rather than with PHs, differing by the specific pathways elicited in treated plants. Our results underline the nature of biostimulants as modulators of specific plant responses, since such responses were not generalized but rather depended on the treatment considered. From a phenotypic point of view, the application of PH alone or in combination with *Trichoderma* had the most positive effect on shoot biomass, followed by the co-inoculation with AMF and *Trichoderma*. The presumed mechanisms behind the desired effects on crop traits by PH or PH + *Trichoderma* could be related (i) to the release of signaling molecules with auxin and ethylene-like activity, in particular bioactive volatile compounds by *Trichoderma* and (ii) to the hormone-like activity (i.e., auxin and gibberellin-like activities) as well as the increase in the activity of key enzymes like glutamine synthetase and nitrate reductase by PH (Schiavon et al., 2008; Vinale et al., 2008; Matsumiya and Kubo, 2011; Colla et al., 2014; Sestili et al., 2018). The former biostimulant activity of PH alone or in combination with *Trichoderma* may have increased nutrient bioavailability to the plants, thus boosting biomass production. On the other hand, the co-inoculation with endophytic fungi stimulated the below ground root growth (higher root biomass) compared to PH, PH + *Trichoderma* and especially to the untreated control. A stimulation/modulation of root auxin production after the inoculation with AMF strains (*Rhizoglomus irregulare* BEG72 and *Funneliformis mosseae* BEG 234) may explain the increase of root biomass in mycorrhized plants, as reported previously in maize, tomato, pepper, lettuce, zucchini, and wheat (Colla et al., 2015a, b; Lucini et al., 2018; Saia et al., 2019, 2020).

The metabolomic profile of both maize leaves and roots were clearly affected by microbial and non-microbial biostimulants, as highlighted by unsupervised and supervised multivariate statistics. As expected, secondary metabolism in both leaves and roots was markedly modulated by the treatments. In general, microorganisms induced a down-regulation of phenylpropanoid, terpenoids, and N-containing secondary metabolites biosynthesis. These findings are supported by the fact that phenylalanine and tryptophan biosynthesis were decreased, although it has been reported that phenylalanine derivates play an important role in plant responses to AMF (Basu et al., 2018). However, in the presence of mycorrhizal fungi, glycolipids and phospholipids, as well as the biosynthetic intermediates of sterols, were up accumulated, mainly in roots. In this sense, several studies suggest that HMG-CoA reductase, a key enzyme of sterol biosynthesis, is involved in plant responses to AMF (MacLean et al., 2017). It is also known that lipids in membranes play an important role in microbial infection and some genes related to lipid metabolism are upregulated in plants following microbial attack. Lipidic metabolites could be involved as signal molecules or by modifying the lipid bilayers. Specifically, lipidic metabolites, such as choline, are involved in the metabolism of phosphatidic acid which is an important secondary messenger. Glycolipids and galactolipids in particular have a relevant function in plant–pathogen interactions and signal transduction (Siebers et al., 2016). The accumulation of these membrane lipids in our plants (mainly in the roots) after the application of microorganisms suggests the involvement of these metabolites in the plants response to microbial biostimulants.

On the other hand, plants treated with PH-containing biostimulants showed a higher number of metabolites involved in the response, as compared to microorganisms alone. Nonetheless, the response of PH and PH + *Trichoderma* were comparable. Despite a direct effect of PH on *Trichoderma* that cannot be excluded *a priori*, our findings suggest a hierarchically stronger effect of PH on plant physiology compared to microorganisms. Considering the pathways affected by PH application, this biostimulant could have a stronger metabolic reprogramming effect by modifying the essential metabolism of N and C. In this sense, it has been reported that PH acts by increasing, either directly or indirectly, the plant growth and crop yield by enhancing nutrient uptake and nutrient use efficiency in plants (Paul et al., 2019a, b). Several studies reveal that PHs stimulated some enzymes involved in N assimilation and C metabolism (Ertani et al., 2009; Colla et al., 2015a, b, 2017b). In our study, PH imposed a marked remodulation of the metabolic pathways of amino acids. Particularly interesting was the upregulation of the phenylalanine biosynthetic pathway after PH application. This aromatic amino acid, biosynthesized *via* shikimate, is a precursor of phenylpropanoids which are linked to plant stress responses (Böttger et al., 2018). Consistently, other metabolites derived from shikimic acid such as chorismate were increased in the presence of PH, in agreement with the concurrent stimulation of the pathway of phenylpropanoid secondary metabolites.

Previous studies support the fact that the phenylpropanoid pathway is part of the reaction of plants to PHs (Lucini et al., 2015, 2018; Carillo et al., 2019). Some biostimulants are postulated to enhance the activity of PAL, a key enzyme in this pathway; Ertani et al. (2017) showed an increase in total phenolic compounds and an increase in the expression of PAL in tomato plants treated with an alfalfa-based PH. Other authors found an increase of PAL (ZmPAL1) gene expression and PAL activity in maize treated with 1 mg C/l of humic substances (Schiavon et al., 2010). These findings corroborate our results, suggesting a crucial role of phenylpropanoids in the response to PH biostimulants.

Hormones were also affected by PH, with a more evident modulation in the roots. The treatments induced reprogramming of the whole phytohormone profile. It is noteworthy that plant growth is known to be regulated by a complex and partially understood interaction network of hormones like auxins, cytokinins, and gibberellins (Liu et al., 2017). In fact, a regulation of all these hormones was triggered in maize roots by the treatments applied in this study, together with the modulation of brassinosteroids and jasmonates. Gibberellins, the most up accumulated phytohormones, play a crucial role in plant development comprising of shoot and root growth, leaf morphogenesis, germination dormancy, seed production, and flowering (Marzec, 2017). DELLA proteins have been related to the interaction between gibberellins and other hormones such as brassinosteroids, jasmonate, and ethylene (Liu et al., 2017). The concurrent increase in abscisic acid following PH treatment is also worth considering, since this hormone coordinates auxins to determine elongation, lateral root formation and architecture in general (Harris, 2015). In relation to hormones, it has been reported that PHs could affect the phytohormonal balance and elicit auxin- and gibberellin-like activities. Moreover, plant bioactive peptides has been noted to have hormone-like activities (Colla et al., 2015a, b, 2017b). In particular, Ertani et al. (2009) and Colla et al. (2014) reported that PHs could have auxin-like and gibberellin-like activity. Although the depiction of this hormonal coordinated network is complicated, we can postulate that the hormonal signatures induced by the treatments are involved in the improved plant growth we observed. The more distinctive phytohormone profile gained in plants treated with the microorganisms alone can also explain the differences in root growth and root-to-shoot ratio we observed from this treatment.

Another factor pertaining to the increased biomass we observed following the biostimulant treatments is the potential involvement of the GS-GOGAT nitrogen assimilation system. Indeed, this system represents the first step of incorporating both ammonium and nitrate into organic compounds. Glutamate and glutamine are the first results of this process, while asparagine is one of the most common ways to export nitrogen to shoots. Consistently, the former accumulated in roots whereas the latter accumulated in shoots. This opens the possibility that the treatments increased nitrogen use efficiency, although specific studies are advisable with this aim. Nonetheless, preliminary evidence that protein hydrolyzates can promote nitrogen use efficiency is present in prior literature (Colla et al., 2017a).

## Conclusion

Over the past decade, the application of microbial and non-microbial biostimulants in intensive and extensive cropping systems has been on the rise, compelled by the increasing interest of growers, scientists, and private sectors. In fact, the agriculture industry has been requested to boost crop productivity in a sustainable manner, even for extensive crops such as maize. A biostimulant activity was observed in the current study in maize, demonstrating that the application of PH alone or in combination with *Trichoderma* as well as the co-inoculation with endophytic fungi (AMF + *Trichoderma*) can generate beneficial effects in terms of plant growth promotion. Co-inoculation with endophytic fungi or the application of PH alone or in combination distinctively modulated the metabolite profile of both maize leaves and roots. Untargeted metabolomics followed by multivariate statistics allowed us to shed light onto the biochemical processes elicited by the treatments. The specific metabolomic signatures achieved from the different treatments indicate that different molecular processes are involved in plant responses to biostimulants, thereby not excluding their combined use in order to provide complementary benefits. Nonetheless, PH induced a hierarchically more pronounced metabolomic response. In general, secondary metabolism (including phenylpropanoids and terpenes) was extensively affected by the different biostimulants, although the direction of regulation was different when only microorganisms (without PH) were used. The multilayer interaction network of phytohormones was also modulated by the treatments, thus supporting the hypothesis of a hormone-like activity ascribed to several biostimulants. For the first time, the treatments considered suggested that nitrogen use efficiency might be involved in the mechanism of the increased plant growth observed. This point is of particular interest and deserves future studies based on more targeted approaches.

## Data Availability Statement

All datasets generated for this study are included in the article/Supplementary Material.

## Author Contributions

YR wrote the first draft of the manuscript, followed the agronomic trial, and contributed to data analysis and interpretation. LL, BM-M, and PB performed the metabolomics analysis, data interpretation, and wrote the metabolomic part. MC and GC were involved in agronomic trial, data analysis, data interpretation, and writing the manuscript. GC and LL coordinated the whole project, provided the intellectual input, set up the experiment, and corrected the manuscript.

## Conflict of Interest

The authors declare that the research was conducted in the absence of any commercial or financial relationships that could be construed as a potential conflict of interest.

## Footnotes

1 http://www.plantcyc.org

2 http://cosmos-fp7.eu/msi.html

## Supplementary Material

TABLE S1 Whole dataset produced from untargeted metabolomic analysis carried out in leaves and roots of maize treated with biostimulants.

TABLE S2 VIP discriminant compounds identified as discriminant from OPLS-DA in leaves following biostimulant application.

TABLE S3 VIP discriminant compounds identified as discriminant from OPLS-DA in roots following biostimulant application.

TABLE S4 Differential metabolites as derived from Volcano analysis (*P*-value < 0.01; FC > 1.2) of leaves following biostimulant application.

TABLE S5 Differential metabolites as derived from Volcano analysis (*P*-value < 0.01; FC > 1.2) of roots following biostimulant application.
